# Interrater and intrarater agreement and reliability of ratings made using the Zaidi–Dayal and Richards–Jabbour scales for the shape of the foramen magnum

**DOI:** 10.1038/s41598-019-54764-0

**Published:** 2019-12-05

**Authors:** Justin Z. Amarin, Sayel H. Alzraikat, Haya H. Suradi, Rand Y. Omari, Afnan N. Ghafel, Darwish H. Badran, Osama A. Samara

**Affiliations:** 1 School of Medicine, The University of Jordan, Amman, Jordan; 2 Department of Radiology, School of Medicine, The University of Jordan, Amman, Jordan; 3 Department of Anatomy and Histology, School of Medicine, The University of Jordan, Amman, Jordan

**Keywords:** Bone, Nervous system

## Abstract

Anatomists and radiologists use the Zaidi–Dayal and Richards–Jabbour scales to study the shape of the foramen magnum. Our aim is to measure the interrater and intrarater agreement and reliability of ratings made using the two scales. We invited 16 radiology residents to attend two sessions, four weeks apart. During each session, we asked the residents to classify the shape of the foramen magnum in 35 images using both scales. We used Fleiss’ κ to measure interrater reliability and Cohen’s κ to measure intrarater reliability. The interrater reliability of ratings made using the Zaidi–Dayal scale was 0.34 (0.26–0.46) for session one and 0.30 (0.24–0.39) for session two, and the intrarater reliability was 0.39 (0.34–0.44). The interrater reliability of ratings made using the Richards–Jabbour scale was 0.14 (0.10–0.19) for session one and 0.12 (0.09–0.17) for session two, and the intrarater reliability was 0.11 (0.07–0.15). In conclusion, the interrater and intrarater agreement and reliability of ratings made using the Zaidi–Dayal and Richards–Jabbour scales are inadequate. We recommend an objective method by Zdilla *et al*. to researchers interested in studying the shape of the foramen magnum.

## Introduction

The foramen magnum is an opening in the occipital bone of the cranium. Vital structures pass through the foramen magnum, including the medulla oblongata, spinal accessory nerves, vertebral arteries, anterior spinal artery, ascending branch of each posterior spinal artery, and posterior meningeal arteries^1^. The size and shape of the foramen magnum in many populations is well-documented^2^.

The size of the foramen magnum is often expressed using the anteroposterior and transverse diameters. The diameters vary by age, biological sex, race, and stature—albeit to a small extent in the normal population^2,3^. Abnormal diameters may indicate pathology: A small foramen magnum occurs with achondroplasia and other skeletal disorders, and a large foramen magnum occurs with chronic increased intracranial pressure, syringomyelia, and Arnold–Chiari malformation. If the structures passing through a small foramen magnum are compressed, a number of clinical symptoms may develop^4^.

The shape of the foramen magnum is often described using one of two nominal scales^5,6^. The scales are used to classify shapes that occur in the normal population. Both scales are appropriate for use in cadaveric and radiographic studies, and the most common users are anatomists and radiologists^2^. The first scale was developed by Zaidi and Dayal and modified by other authors to include seven categories: “round”, “oval”, “egg-shaped”, “tetragonal”, “pentagonal”, “hexagonal”, and “irregular”^5^. The second scale was developed by Richards and Jabbour and includes eight categories: “circular”, “two semicircles”, “heart-like”, “wide oval”, “bi-rounded oval”, “ventrally wide oval”, “bi-pointed oval”, and “dorsally convergent oval”^6^. Abnormal shapes may indicate pathology: An asymmetric foramen magnum occurs with craniovertebral junction anomalies and craniosynostosis, and a keyhole-shaped foramen magnum occurs with hydrolethalus syndrome^4^. In addition, surgical recommendations have been made based on the shape of the foramen magnum^7^.

The shape of the foramen magnum varies to a large extent between populations^2^. The variability may be explained by true population variability or measurement error. The measurement errors of the Zaidi–Dayal and Richards–Jabbour scales have not been quantified. Our aim is to measure the interrater and intrarater agreement and reliability of ratings made using the two scales.

## Methods

### Images

Previously, we described the shape of the foramen magnum in 247 images of the cranial base, captured by computed tomography^3^. We had classified the images into the categories of the Zaidi–Dayal scale^5^. For our current study, we selected 35 out of 247 images by stratified random sampling using an online true random number service based on atmospheric noise^8^. We sampled five images per stratum, with each stratum representing one out of the seven categories of the scale. From each image, we captured a 200 × 200 pixels snip of the foramen magnum with a rough circumferential margin of 40 pixels and saved the snips as Portable Network Graphics (.png) files. We created two presentations using Google Slides and populated each presentation with the 35 snips (one per slide). In each presentation, we sorted the slides according to a random sequence generated using an online true random number service based on atmospheric noise^8^. We set the slides to auto-advance every minute.

### Raters

We recruited our raters by purposive sampling. The Department of Radiology (School of Medicine, The University of Jordan) offers an approximate total of 20 residency positions. During the study period, 16 residents were active. We invited all active residents to participate in our study. The residents were naïve to both the Zaidi–Dayal and Richards–Jabbour scales. The Institutional Review Board of the Jordan University Hospital (Amman, Jordan) approved the study protocol, and all participants provided written informed consent. We conducted the study according to the 1964 Declaration of Helsinki and subsequent amendments.

### Procedure

We asked the raters to attend two sessions. The time interval between the sessions was four weeks. We conducted the sessions in the radiology conference room (Jordan University Hospital). Each session included a training period (∼10 minutes) and a rating period (35 minutes). Before each session, we distributed document packets containing a data collection form, two answer sheets, and a reference sheet. We asked the raters to fill out the data collection form before the training period. During the training period, we introduced (session one) or reintroduced (session two) both scales to the raters and presented an example—from the published literature—of each category^5,6^. The information was available on the reference sheet throughout the entire session. During the rating period, we displayed the presentation on a projection screen and asked the raters to fill out two answer sheets (one per scale). We ran the rating period under exam conditions to ensure independent classification. We made no attempt to control the Hawthorne effect because optimal performance is conducive to the validity of our results.

### Power analysis

We used R (version 3.6.1) to perform power analysis. Rotondi and Donner developed a confidence interval approach for sample size estimation in reliability studies and implemented the method in kappaSize, an R software package^9^. The functions of the package are limited to a maximum of five categories and six raters. To estimate the minimum number of images, we called the CI5Cats function of the kappaSize package (version 1.2). The arguments of the function are the anticipated κ, the desired confidence interval, the relative frequency of each category, the number of raters, and the type I error rate. The estimate decreases with a greater number of categories, a greater anticipated κ, a wider desired confidence interval, unequal relative frequencies, a greater number of raters, and a higher type I error rate^9^. We set the anticipated κ to 0.4 and the desired confidence interval to 0.2–0.6. The values correspond to minimal-to-weak reliability according to McHugh^10^. We assumed equal relative frequencies because we selected the images by stratified random sampling. We set the number of raters to two because intrarater reliability is measured for two sets of ratings. Finally, we set the type I error rate to 0.05. The estimated minimum number of images was 35. The estimate is inflated because we used a minimum of seven categories, not five (the maximum limit of the function).

### Data validation and analysis

Using Google Sheets, we manually entered the data twice into separate sheets and compared them using built-in functions to detect data entry errors. We saved the validated data set as a Comma-Separated Values (.CSV) file and imported the file into R (version 3.6.1), which we used to perform data analysis. To measure interrater reliability, we stratified the data by session (session one or session two) and scale (Zaidi–Dayal or Richards–Jabbour) and computed Fleiss’ κ for each of the four data subsets using the kappam.fleiss function of the irr package (version 0.84.1). To measure intrarater reliability, we stratified the data by rater and scale and computed Cohen’s κ for each of the 32 data subsets using the kappa2 function of the irr package (version 0.84.1). To measure the uncertainty of Fleiss’ κ, we computed the standard error of the statistic from 1,000 ordinary nonparametric bootstrap replicates using the boot function of the boot package (version 1.3.23). Then, we computed the bias-corrected and accelerated confidence interval using the boot.ci function of the boot package (version 1.3.23). To measure the uncertainty of Cohen’s κ, we computed the standard error of the statistic from 1,000 ordinary bootstrap replicates using the boot function of the boot package (version 1.3.23). We pooled the point estimates and standard errors of Cohen’s κ for each scale using the generic inverse variance method implemented in the metagen function of the meta package (version 4.9.6). We present numerical data according to the recommendations of Cole^11^. We report point estimates of κ alongside 95% confidence intervals (the latter within parentheses), absolute frequencies alongside relative frequencies (the latter within parentheses), and medians alongside ranges (the latter within parentheses).

## Results

### Rater characteristics

We invited 16 radiology residents to participate in our study, and all of them accepted. The median age of the raters was 27 years (24–32 years). Two raters (12%) were male and 14 (88%) were female. Seven raters (44%) were first-year residents, five (31%) were second-year residents, and four (25%) were fourth-year residents. All raters attended both sessions and completed *N* = 2,240 ratings.

### Interrater reliability

The observed proportion of majority interrater agreement is the proportion of images that were classified into a particular category by at least nine out of 16 raters—the majority. For ratings made using the Zaidi–Dayal scale, the observed proportion was 63% in session one and 57% in session two. For ratings made using the Richards–Jabbour scale, the observed proportion was 23% in both sessions. The proportion of majority interrater agreement expected by pure chance is 0.08% and 0.03% for the Zaidi–Dayal and the Richards–Jabbour scales, respectively. We measured interrater reliability using Fleiss’ κ. The interrater reliability of ratings made using the Zaidi–Dayal scale was 0.34 (0.26–0.46) for session one and 0.30 (0.24–0.39) for session two. Both estimates indicate minimal-to-weak reliability. On the other hand, the interrater reliability of ratings made using the Richards–Jabbour scale was 0.14 (0.10–0.19) for session one and 0.12 (0.09–0.17) for session two. Both estimates indicate no reliability. We report the category-wise interrater reliability of ratings made using each scale in Table 1. We present the images and image-wise ratings in the Supplementary Document.

**Table 1:** Category-wise interrater reliability of ratings made using the Zaidi–Dayal and Richards–Jabbour scales.

Category	Classification rate	Interrater reliability (Fleiss’ κ)	
		Session one	Session two
**Zaidi–Dayal scale**			
Round	6%	0.23	0.20
Oval	18%	0.25	0.34
Egg-shaped	16%	0.23	0.16
Tetragonal	13%	0.42	0.28
Pentagonal	10%	0.36	0.22
Hexagonal	22%	0.47	0.50
Irregular	16%	0.33	0.29
**Richards–Jabbour scale**			
Circular	7%	0.19	0.19
Two semicircles	15%	0.08	0.08
Heart-like	12%	0.24	0.33
Wide oval	10%	0.10	0.04
Bi-rounded oval	12%	0.04	0.05
Ventrally wide oval	11%	0.07	0.03
Bi-pointed oval	21%	0.20	0.19
Dorsally convergent oval	13%	0.16	0.05

### Optimal reliability

One way to modify a nominal scale is to form one or more supercategories. A supercategory is a set of two or more categories. For example, a seven-categories scale may be converted into a two-categories scale by retaining one original category and combining the remainder. The total number of ways to modify seven- and eight-categories scales by forming one or more supercategories is 877 and 4140, respectively. We iterated the analysis over the entire range using a custom Python script to find the version of each scale that optimizes the interrater reliability of the collected data. We present the optimal *n*-categories solutions in Table 2.

**Table 2:** Optimal interrater reliability of ratings made using the Zaidi–Dayal and Richards–Jabbour scales.

Zaidi–Dayal scale		Session one	Session two
Optimal two-categories solution	Category one	“Round”, “Oval”, “Egg-shaped”, “Tetragonal”, or “Irregular”	“Round”, “Oval”, “Egg-shaped”, “Tetragonal”, “Pentagonal”, or “Irregular”
	Category two	“Pentagonal” or “Hexagonal”	“Hexagonal”
	Fleiss’ κ (95% CI)	0.49 (0.38–0.63)	0.50 (0.37–0.65)
Optimal three-categories solution	Category one	“Round”, “Oval”, “Egg-shaped”, “Tetragonal”, or “Irregular”	“Round”
	Category two	“Pentagonal”	“Oval”, “Egg-shaped”, “Tetragonal”, “Pentagonal”, or “Irregular”
	Category three	“Hexagonal”	“Hexagonal”
	Fleiss’ κ (95% CI)	0.46 (0.36–0.58)	0.43 (0.33–0.56)
Optimal four-categories solution	Category one	“Round”	“Round”
	Category two	“Oval”, “Egg-shaped”, “Tetragonal”, or “Irregular”	“Oval”, “Egg-shaped”, “Tetragonal”, “Pentagonal”
	Category three	“Pentagonal”	“Hexagonal”
	Category four	“Hexagonal”	“Irregular”
	Fleiss’ κ (95% CI)	0.42 (0.34–0.54)	0.40 (0.31–0.53)
Optimal five-categories solution	Category one	“Round”	“Round”
	Category two	“Oval”, “Egg-shaped”, or “Tetragonal”	“Oval”, “Egg-shaped”, or “Tetragonal”
	Category three	“Pentagonal”	“Pentagonal”
	Category four	“Hexagonal”	“Hexagonal”
	Category five	“Irregular”	“Irregular”
	Fleiss’ κ (95% CI)	0.40 (0.32–0.54)	0.37 (0.27–0.47)
Optimal six-categories solution	Category one	“Round”	“Round”
	Category two	“Oval” or “Egg-shaped”	“Oval” or “Egg-shaped”
	Category three	“Tetragonal”	“Tetragonal”
	Category four	“Pentagonal”	“Pentagonal”
	Category five	“Hexagonal”	“Hexagonal”
	Category six	“Irregular”	“Irregular”
	Fleiss’ κ (95% CI)	0.37 (0.29–0.46)	0.33 (0.25–0.42)
**Richards–Jabbour scale**		**Session one**	**Session two**
Optimal two-categories solution	Category one	Circular, “Two semicircles”, “Wide oval”, “Bi-rounded oval”, or “Bi-pointed oval”	Circular, “Two semicircles”, “Wide oval”, “Bi-rounded oval”, “Ventrally wide oval”, “Bi-pointed oval”, or “Dorsally convergent oval”
	Category two	“Heart-like”, “Ventrally wide oval”, or “Dorsally convergent oval”	“Heart-like”
	Fleiss’ κ (95% CI)	0.30 (0.18–0.49)	0.33 (0.19–0.45)
Optimal three-categories solution	Category one	Circular	Circular
	Category two	“Two semicircles”, “Wide oval”, “Bi-rounded oval”, or “Bi-pointed oval”	“Two semicircles”, “Wide oval”, “Bi-rounded oval”, “Ventrally wide oval”, “Bi-pointed oval”, or “Dorsally convergent oval”
	Category three	“Heart-like”, “Ventrally wide oval”, or “Dorsally convergent oval”	“Heart-like”
	Fleiss’ κ (95% CI)	0.27 (0.16–0.41)	0.26 (0.16–0.37)
Optimal four-categories solution	Category one	Circular	Circular, “Two semicircles”, or “Ventrally wide oval”
	Category two	“Two semicircles”, “Wide oval”, “Bi-rounded oval”, or “Bi-pointed oval”	“Heart-like”
	Category three	“Heart-like” or “Dorsally convergent oval”	“Wide oval”, “Bi-rounded oval”, or “Bi-pointed oval”
	Category four	“Ventrally wide oval”	“Dorsally convergent oval”
	Fleiss’ κ (95% CI)	0.23 (0.16–0.32)	0.22 (0.15–0.34)
Optimal five-categories solution	Category one	Circular	Circular, “Two semicircles”, or “Ventrally wide oval”
	Category two	“Two semicircles”, “Wide oval”, “Bi-rounded oval”, or “Bi-pointed oval”	“Heart-like”
	Category three	“Heart-like”	“Wide oval”
	Category four	“Ventrally wide oval”	“Bi-rounded oval” or “Bi-pointed oval”
	Category five	“Dorsally convergent oval”	“Dorsally convergent oval”
	Fleiss’ κ (95% CI)	0.20 (0.14–0.28)	0.19 (0.14–0.28)
Optimal six-categories solution	Category one	Circular, “Two semicircles”, or “Wide oval”	Circular, “Two semicircles”, or “Ventrally wide oval”
	Category two	“Heart-like”	“Heart-like”
	Category three	“Bi-rounded oval”	“Wide oval”
	Category four	“Ventrally wide oval”	“Bi-rounded oval”
	Category five	“Bi-pointed oval”	“Bi-pointed oval”
	Category six	“Dorsally convergent oval”	“Dorsally convergent oval”
	Fleiss’ κ (95% CI)	0.18 (0.13–0.26)	0.17 (0.13–0.26)
Optimal seven-categories solution	Category one	Circular	Circular or “Two semicircles”
	Category two	“Two semicircles”	“Heart-like”
	Category three	“Heart-like” or “Dorsally convergent oval”	“Wide oval”
	Category four	“Wide oval”	“Bi-rounded oval”
	Category five	“Bi-rounded oval”	“Ventrally wide oval”
	Category six	“Ventrally wide oval”	“Bi-pointed oval”
	Category seven	“Bi-pointed oval”	“Dorsally convergent oval”
	Fleiss’ κ (95% CI)	0.16 (0.11–0.20)	0.14 (0.10–0.21)

### Intrarater reliability

The observed proportion of intrarater agreement is the proportion of images that were classified into a particular category twice by the same rater. For ratings made using the Zaidi–Dayal scale, the median observed proportion of intrarater agreement was 50% (26–74%). For ratings made using the Richards–Jabbour scale, the median observed proportion of intrarater agreement was 29% (9–40%). The proportion of intrarater agreement expected by pure chance is 14% and 13% for ratings made using the Zaidi–Dayal and Richards–Jabbour scales, respectively. We measured intrarater reliability using Cohen’s κ. The intrarater reliability of ratings made using the Zaidi–Dayal scale was 0.39 (0.34–0.44), which indicates minimal-to-weak reliability. The intrarater reliability of ratings made using the Richards–Jabbour scale was 0.11 (0.07–0.15), which indicates no intrarater reliability.

## Discussion

We recruited radiology residents to rate the shape of the foramen magnum using the Zaidi–Dayal and Richards–Jabbour scales. Then, we estimated the interrater and intrarater agreement and reliability of the ratings made using each scale. The estimates were higher than those expected by pure chance. However, the estimates of interrater and intrarater reliability were lower than 0.6, which is the minimum value that indicates “adequate” reliability^10^.

The frequency distribution of the shapes of the foramen magnum varies widely from study to study^2,3^. The variability may result from true population variability or measurement error. True population variability is more likely if κ estimates indicate high reliability, while measurement error is more likely if κ estimates indicate low reliability. To interpret κ estimates, we have the choice between many guidelines—none of which are standard, and most of which are arbitrary^12^. Ultimately, we chose to interpret our results according to McHugh’s guidelines, which are substantiated, actionable, and widely accepted^10^. According to McHugh’s guidelines, our results indicate that little confidence should be placed in ratings made using the Zaidi–Dayal and Richards–Jabbour scales. Therefore, measurement error explains, at least in part, the variable frequency distribution of the shapes of the foramen magnum between populations.

κ estimates may increase or decrease if the categories of a scale are combined^13–15^. The change depends on the interaction between the raters and the scale. For example, if raters often confuse two categories, the κ estimate would increase if the categories are combined. Using a computational method, we attempted to find the versions of each scale that coax the data into yielding optimal κ estimates. None of the optimal κ estimates was equal to or higher than 0.6—the McHugh threshold. Our results suggest that neither scale may be “salvaged” by forming supercategories.

Complex relationships between points on the outline of the foramen magnum determine the shape of the foramen magnum. The relationships are governed by developmental and environmental factors^16^. Therefore, the shape of the foramen magnum is a complex construct. Our raters measured the construct on two nominal scales by qualitative observation. The method is accessible but uniparametric, limited, and subjective. In a recent article, Zdilla *et al*. developed an alternative method. They used an image processing software to measure four dimensions of the construct on a continuous scale^16^. The Zdilla *et al*. method is multiparametric, flexible, and objective, but requires technical skill. The Zdilla *et al*. method must be streamlined to encourage the disuse of methods that rely on nominal scales. In any case, we recommend the Zdilla *et al*. method for shape analysis in future studies of the foramen magnum.

Our results must be interpreted with caution because our sampling methods are limited. We recruited a nonrandom sample of raters who represent a part of the population of raters. However, our sample is homogeneous, and homogeneity inflates agreement and reliability. In addition, our results should not be used to compare the Zaidi–Dayal and Richards–Jabbour scales head-to-head because the images were sampled across the categories of the Zaidi–Dayal scale, not the Richards–Jabbour scale. In addition, the Zaidi–Dayal scale features a “throwaway” category (“Irregular”), while the Richards–Jabbour scale does not. A “throwaway” category translates uncertainty into agreement and reliability and inflates the estimates. However, the distribution of ratings across the categories of both scales is roughly uniform and suggests that neither source of bias is influential. Overall, the limitations do not compromise our conclusions because they bias the estimates toward the opposite direction.

In conclusion, the interrater and intrarater agreement and reliability of ratings made using the Zaidi–Dayal and Richards–Jabbour scales are inadequate. We recommend the Zdilla *et al*. method to researchers interested in studying the shape of the foramen magnum.

## Supplementary information

Supplementary Document

## Data Availability

The authors will make the data available upon request.
