# Supplementary material for: Interrater and intrarater agreement and reliability of ratings made using the Zaidi–Dayal and Richards–Jabbour scales for the shape of the foramen magnum
Source: Sci Rep. 2019 Dec 5;9:18384. doi: 10.1038/s41598-019-54764-0 (PMC6895155; doi:10.1038/s41598-019-54764-0)
Supplement: Supplementary file 1 — Supplementary Document [file 41598_2019_54764_MOESM1_ESM.pdf]

Supplementary Document

Interrater and intrarater agreement and reliability of ratings made using the Zaidi–Dayal and Richards–Jabbour scales for the shape of the foramen magnum

Justin Z. Amarin, Sayel H. Alzraikat, Haya H. Suradi, Rand Y. Omari, Afnan N. Ghafel, Darwish H. Badran, Osama A. Samara

Synopsis

We asked 16 raters to classify the shape of the foramen magnum in 35 images using the Zaidi–Dayal and Richards–Jabbour scales. We present the images and image-wise ratings below. Our ratings—from a previous study—are highlighted in purple.

1

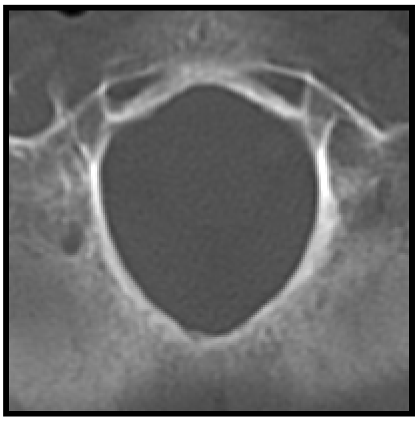

Category	Ratings • *n* (%)	
	Session one	Session two
**Zaidi–Dayal scale**		
Round	0 (0)	0 (0)
Oval	**6 (38)**	2 (13)
Egg-shaped	2 (13)	4 (25)
Tetragonal	2 (13)	1 (6)
Pentagonal	1 (6)	3 (19)
Hexagonal	0 (0)	1 (6)
Irregular	5 (31)	**5 (31)**
**Richards–Jabbour scale**		
Circular	0 (0)	0 (0)
Two semicircles	1 (6)	1 (6)
Heart-like	**8 (50)**	**10 (63)**
Wide oval	0 (0)	1 (6)
Bi-rounded oval	0 (0)	1 (6)
Ventrally wide oval	4 (25)	0 (0)
Bi-pointed oval	0 (0)	1 (6)
Dorsally convergent oval	3 (19)	2 (13)

2

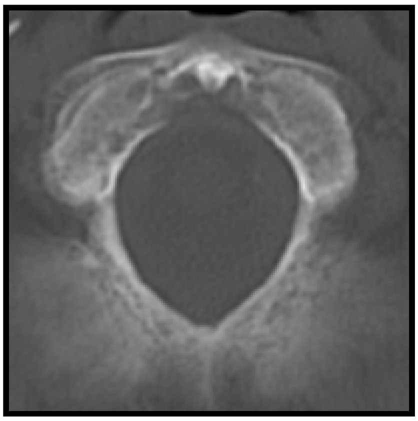

Category	Ratings • *n* (%)	
	Session one	Session two
**Zaidi–Dayal scale**		
Round	0 (0)	0 (0)
Oval	**7 (44)**	**9 (56)**
Egg-shaped	2 (13)	3 (19)
Tetragonal	5 (31)	4 (25)
Pentagonal	0 (0)	0 (0)
Hexagonal	0 (0)	0 (0)
Irregular	2 (13)	0 (0)
**Richards–Jabbour scale**		
Circular	0 (0)	1 (6)
Two semicircles	0 (0)	2 (13)
Heart-like	2 (13)	3 (19)
Wide oval	0 (0)	0 (0)
Bi-rounded oval	0 (0)	2 (13)
Ventrally wide oval	2 (13)	2 (13)
Bi-pointed oval	1 (6)	**4 (25)**
Dorsally convergent oval	**11 (69)**	2 (13)

3

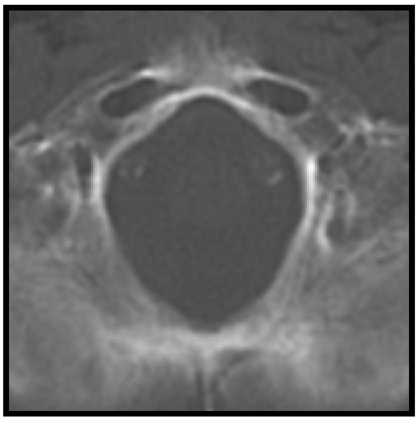

Category	Ratings • *n* (%)	
	**Session one**	**Session two**
**Zaidi–Dayal scale**		
Round	0 (0)	0 (0)
Oval	2 (13)	1 (6)
Egg-shaped	2 (13)	4 (25)
Tetragonal	**10 (63)**	**7 (44)**
Pentagonal	0 (0)	2 (13)
Hexagonal	1 (6)	0 (0)
Irregular	1 (6)	2 (13)
**Richards–Jabbour scale**		
Circular	0 (0)	0 (0)
Two semicircles	0 (0)	0 (0)
Heart-like	6 (38)	**10 (63)**
Wide oval	0 (0)	1 (6)
Bi-rounded oval	1 (6)	4 (25)
Ventrally wide oval	0 (0)	0 (0)
Bi-pointed oval	**8 (50)**	0 (0)
Dorsally convergent oval	1 (6)	1 (6)

4

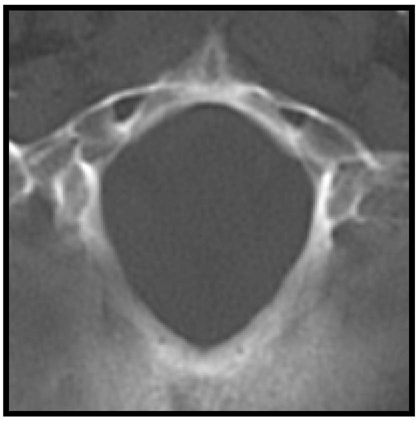

Category	Ratings • *n* (%)	
	Session one	Session two
**Zaidi–Dayal scale**		
Round	0 (0)	0 (0)
Oval	0 (0)	2 (13)
Egg-shaped	3 (19)	**6 (38)**
Tetragonal	**10 (63)**	5 (31)
Pentagonal	0 (0)	0 (0)
Hexagonal	0 (0)	1 (6)
Irregular	3 (19)	2 (13)
**Richards–Jabbour scale**		
Circular	0 (0)	0 (0)
Two semicircles	0 (0)	0 (0)
Heart-like	5 (31)	**12 (75)**
Wide oval	0 (0)	0 (0)
Bi-rounded oval	0 (0)	1 (6)
Ventrally wide oval	3 (19)	0 (0)
Bi-pointed oval	1 (6)	0 (0)
Dorsally convergent oval	**7 (44)**	3 (19)

5

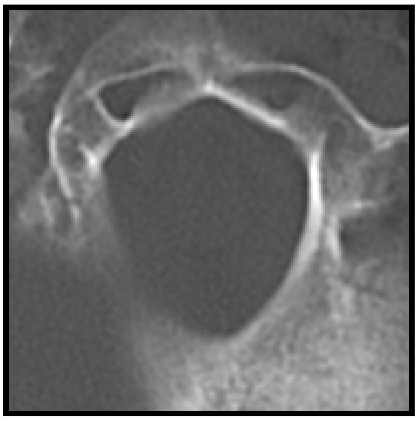

Category	Ratings • *n* (%)	
	Session one	Session two
**Zaidi–Dayal scale**		
Round	0 (0)	0 (0)
Oval	1 (6)	3 (19)
Egg-shaped	3 (19)	2 (13)
Tetragonal	**7 (44)**	**4 (25)**
Pentagonal	3 (19)	3 (19)
Hexagonal	0 (0)	0 (0)
Irregular	2 (13)	**4 (25)**
**Richards–Jabbour scale**		
Circular	0 (0)	0 (0)
Two semicircles	0 (0)	1 (6)
Heart-like	**12 (75)**	**9 (56)**
Wide oval	0 (0)	0 (0)
Bi-rounded oval	1 (6)	1 (6)
Ventrally wide oval	0 (0)	1 (6)
Bi-pointed oval	3 (19)	3 (19)
Dorsally convergent oval	0 (0)	1 (6)

6

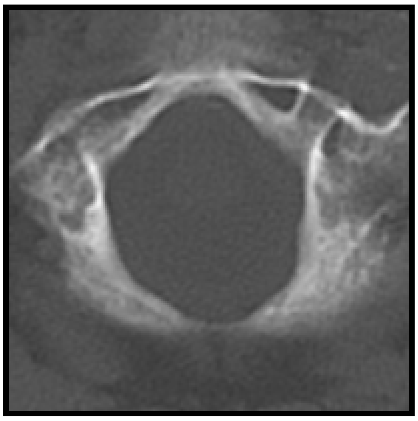

Category	Ratings • *n* (%)	
	Session one	Session two
**Zaidi–Dayal scale**		
Round	0 (0)	0 (0)
Oval	0 (0)	0 (0)
Egg-shaped	1 (6)	0 (0)
Tetragonal	0 (0)	1 (6)
Pentagonal	3 (19)	2 (13)
Hexagonal	**11 (69)**	**12 (75)**
Irregular	1 (6)	1 (6)
**Richards–Jabbour scale**		
Circular	0 (0)	0 (0)
Two semicircles	0 (0)	1 (6)
Heart-like	0 (0)	0 (0)
Wide oval	0 (0)	2 (13)
Bi-rounded oval	3 (19)	**6 (38)**
Ventrally wide oval	2 (13)	1 (6)
Bi-pointed oval	**10 (63)**	4 (25)
Dorsally convergent oval	1 (6)	2 (13)

7

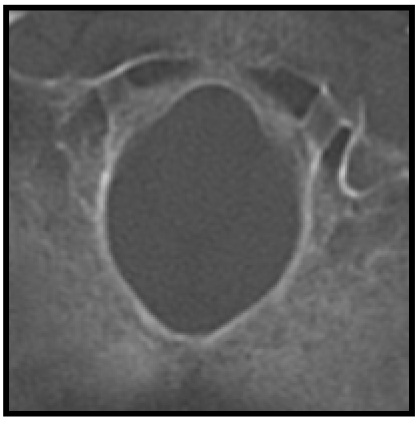

Category	Ratings • *n* (%)	
	Session one	Session two
**Zaidi–Dayal scale**		
Round	0 (0)	0 (0)
Oval	**9 (56)**	**10 (63)**
Egg-shaped	4 (25)	5 (31)
Tetragonal	0 (0)	1 (6)
Pentagonal	0 (0)	0 (0)
Hexagonal	2 (13)	0 (0)
Irregular	1 (6)	0 (0)
**Richards–Jabbour scale**		
Circular	0 (0)	0 (0)
Two semicircles	3 (19)	3 (19)
Heart-like	0 (0)	2 (13)
Wide oval	3 (19)	2 (13)
Bi-rounded oval	**9 (56)**	**5 (31)**
Ventrally wide oval	0 (0)	1 (6)
Bi-pointed oval	0 (0)	2 (13)
Dorsally convergent oval	1 (6)	1 (6)

8

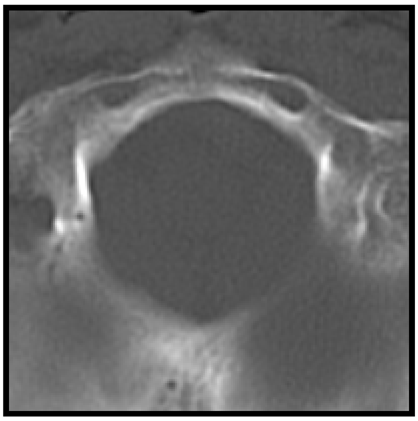

Category	Ratings • *n* (%)	
	**Session one**	**Session two**
**Zaidi–Dayal scale**		
Round	5 (31)	1 (6)
Oval	0 (0)	0 (0)
Egg-shaped	0 (0)	0 (0)
Tetragonal	1 (6)	0 (0)
Pentagonal	2 (13)	1 (6)
Hexagonal	**8 (50)**	**13 (81)**
Irregular	0 (0)	1 (6)
**Richards–Jabbour scale**		
Circular	3 (19)	2 (13)
Two semicircles	1 (6)	**4 (25)**
Heart-like	0 (0)	1 (6)
Wide oval	1 (6)	1 (6)
Bi-rounded oval	1 (6)	1 (6)
Ventrally wide oval	3 (19)	**4 (25)**
Bi-pointed oval	0 (0)	2 (13)
Dorsally convergent oval	**7 (44)**	1 (6)

9

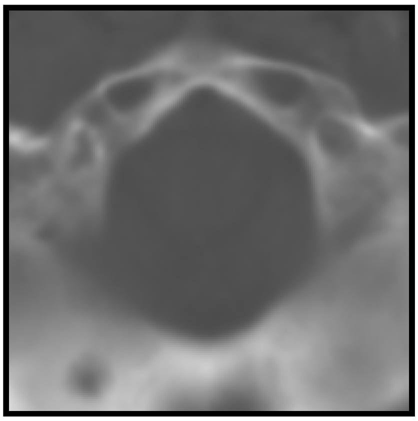

Category	Ratings • *n* (%)	
	Session one	Session two
**Zaidi–Dayal scale**		
Round	0 (0)	0 (0)
Oval	0 (0)	0 (0)
Egg-shaped	3 (19)	3 (19)
Tetragonal	0 (0)	0 (0)
Pentagonal	1 (6)	2 (13)
Hexagonal	**12 (75)**	**11 (69)**
Irregular	0 (0)	0 (0)
**Richards–Jabbour scale**		
Circular	0 (0)	0 (0)
Two semicircles	4 (25)	3 (19)
Heart-like	0 (0)	0 (0)
Wide oval	0 (0)	1 (6)
Bi-rounded oval	1 (6)	2 (13)
Ventrally wide oval	1 (6)	0 (0)
Bi-pointed oval	**9 (56)**	**9 (56)**
Dorsally convergent oval	1 (6)	1 (6)

10

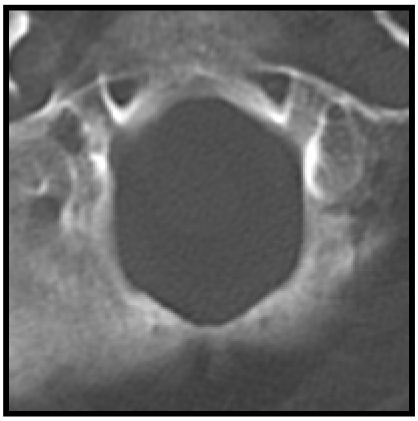

Category	Ratings • *n* (%)	
	Session one	Session two
**Zaidi–Dayal scale**		
Round	0 (0)	0 (0)
Oval	0 (0)	0 (0)
Egg-shaped	0 (0)	0 (0)
Tetragonal	0 (0)	0 (0)
Pentagonal	0 (0)	0 (0)
Hexagonal	**16 (100)**	**15 (94)**
Irregular	0 (0)	1 (6)
**Richards–Jabbour scale**		
Circular	0 (0)	0 (0)
Two semicircles	2 (13)	1 (6)
Heart-like	0 (0)	0 (0)
Wide oval	0 (0)	1 (6)
Bi-rounded oval	4 (25)	4 (25)
Ventrally wide oval	0 (0)	2 (13)
Bi-pointed oval	**9 (56)**	**8 (50)**
Dorsally convergent oval	1 (6)	0 (0)

11

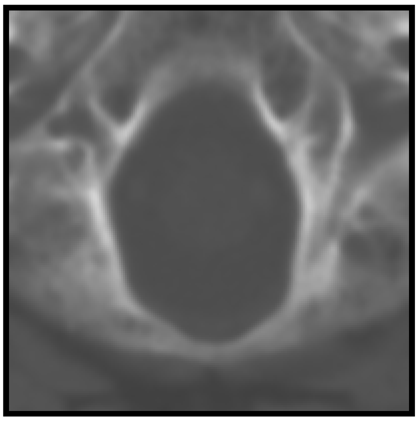

Category	Ratings • *n* (%)	
	Session one	Session two
**Zaidi–Dayal scale**		
Round	0 (0)	0 (0)
Oval	1 (6)	5 (31)
Egg-shaped	1 (6)	1 (6)
Tetragonal	0 (0)	0 (0)
Pentagonal	1 (6)	0 (0)
Hexagonal	**11 (69)**	**7 (44)**
Irregular	2 (13)	3 (19)
**Richards–Jabbour scale**		
Circular	0 (0)	0 (0)
Two semicircles	0 (0)	0 (0)
Heart-like	0 (0)	1 (6)
Wide oval	0 (0)	4 (25)
Bi-rounded oval	2 (13)	**6 (38)**
Ventrally wide oval	3 (19)	1 (6)
Bi-pointed oval	**9 (56)**	2 (13)
Dorsally convergent oval	2 (13)	2 (13)

12

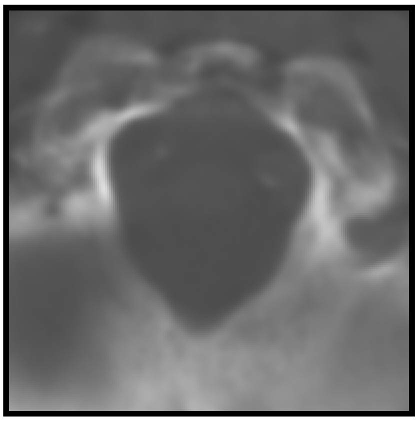

Category	Ratings • *n* (%)	
	Session one	Session two
**Zaidi–Dayal scale**		
Round	0 (0)	0 (0)
Oval	0 (0)	0 (0)
Egg-shaped	1 (6)	0 (0)
Tetragonal	1 (6)	0 (0)
Pentagonal	0 (0)	0 (0)
Hexagonal	**12 (75)**	**9 (56)**
Irregular	2 (13)	7 (44)
**Richards–Jabbour scale**		
Circular	0 (0)	0 (0)
Two semicircles	0 (0)	0 (0)
Heart-like	**6 (38)**	6 (38)
Wide oval	0 (0)	0 (0)
Bi-rounded oval	3 (19)	**8 (50)**
Ventrally wide oval	0 (0)	0 (0)
Bi-pointed oval	2 (13)	0 (0)
Dorsally convergent oval	5 (31)	2 (13)

13

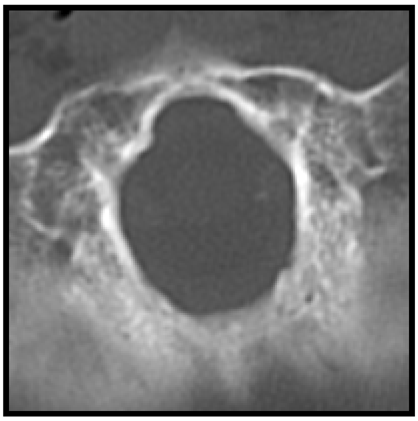

Category	Ratings • *n* (%)	
	Session one	Session two
**Zaidi–Dayal scale**		
Round	0 (0)	0 (0)
Oval	0 (0)	0 (0)
Egg-shaped	0 (0)	0 (0)
Tetragonal	0 (0)	0 (0)
Pentagonal	0 (0)	0 (0)
Hexagonal	0 (0)	0 (0)
Irregular	**16 (100)**	**16 (100)**
**Richards–Jabbour scale**		
Circular	1 (6)	2 (13)
Two semicircles	0 (0)	2 (13)
Heart-like	1 (6)	**4 (25)**
Wide oval	1 (6)	2 (13)
Bi-rounded oval	**7 (44)**	1 (6)
Ventrally wide oval	5 (31)	2 (13)
Bi-pointed oval	1 (6)	1 (6)
Dorsally convergent oval	0 (0)	2 (13)

14

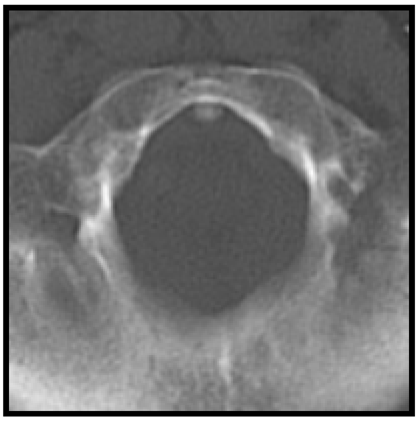

Category	Ratings • *n* (%)	
	Session one	Session two
**Zaidi–Dayal scale**		
Round	0 (0)	0 (0)
Oval	0 (0)	0 (0)
Egg-shaped	0 (0)	0 (0)
Tetragonal	1 (6)	1 (6)
Pentagonal	0 (0)	0 (0)
Hexagonal	**10 (63)**	**12 (75)**
Irregular	5 (31)	3 (19)
**Richards–Jabbour scale**		
Circular	2 (13)	0 (0)
Two semicircles	1 (6)	**4 (25)**
Heart-like	1 (6)	1 (6)
Wide oval	0 (0)	1 (6)
Bi-rounded oval	**4 (25)**	1 (6)
Ventrally wide oval	1 (6)	2 (13)
Bi-pointed oval	**4 (25)**	**4 (25)**
Dorsally convergent oval	3 (19)	3 (19)

15

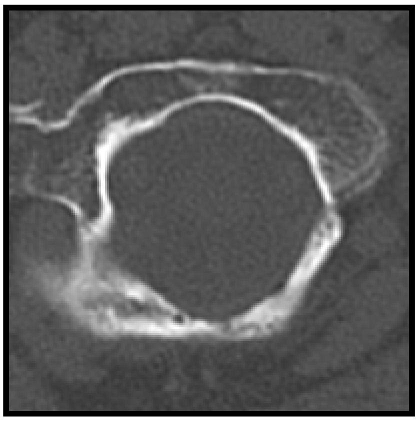

Category	Ratings • *n* (%)	
	Session one	Session two
**Zaidi–Dayal scale**		
Round	4 (25)	2 (13)
Oval	0 (0)	0 (0)
Egg-shaped	0 (0)	0 (0)
Tetragonal	0 (0)	0 (0)
Pentagonal	0 (0)	1 (6)
Hexagonal	1 (6)	0 (0)
Irregular	**11 (69)**	**13 (81)**
**Richards–Jabbour scale**		
Circular	**8 (50)**	5 (31)
Two semicircles	1 (6)	**6 (38)**
Heart-like	3 (19)	1 (6)
Wide oval	2 (13)	0 (0)
Bi-rounded oval	1 (6)	0 (0)
Ventrally wide oval	0 (0)	4 (25)
Bi-pointed oval	1 (6)	0 (0)
Dorsally convergent oval	0 (0)	0 (0)

16

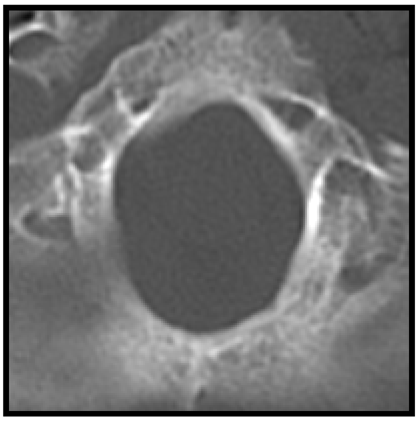

Category	Ratings • *n* (%)	
	Session one	Session two
**Zaidi–Dayal scale**		
Round	1 (6)	1 (6)
Oval	0 (0)	0 (0)
Egg-shaped	1 (6)	**7 (44)**
Tetragonal	0 (0)	0 (0)
Pentagonal	**7 (44)**	1 (6)
Hexagonal	5 (31)	1 (6)
Irregular	2 (13)	6 (38)
**Richards–Jabbour scale**		
Circular	0 (0)	0 (0)
Two semicircles	**4 (25)**	**5 (31)**
Heart-like	0 (0)	0 (0)
Wide oval	**4 (25)**	3 (19)
Bi-rounded oval	3 (19)	1 (6)
Ventrally wide oval	1 (6)	**5 (31)**
Bi-pointed oval	**4 (25)**	0 (0)
Dorsally convergent oval	0 (0)	2 (13)

17

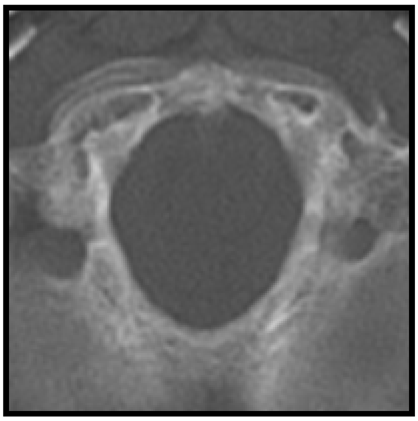

Category	Ratings • *n* (%)	
	Session one	Session two
**Zaidi–Dayal scale**		
Round	0 (0)	1 (6)
Oval	**10 (63)**	**11 (69)**
Egg-shaped	1 (6)	1 (6)
Tetragonal	2 (13)	2 (13)
Pentagonal	2 (13)	0 (0)
Hexagonal	1 (6)	0 (0)
Irregular	0 (0)	1 (6)
**Richards–Jabbour scale**		
Circular	0 (0)	1 (6)
Two semicircles	4 (25)	**6 (38)**
Heart-like	1 (6)	2 (13)
Wide oval	1 (6)	2 (13)
Bi-rounded oval	4 (25)	0 (0)
Ventrally wide oval	**6 (38)**	4 (25)
Bi-pointed oval	0 (0)	1 (6)
Dorsally convergent oval	0 (0)	0 (0)

18

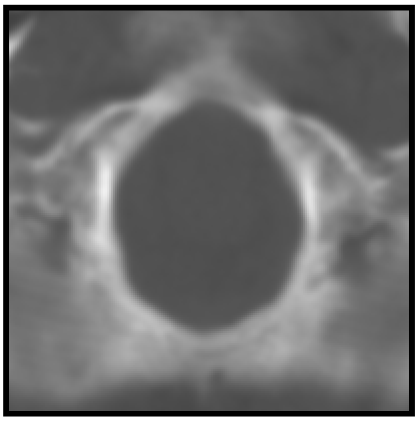

Category	Ratings • *n* (%)	
	Session one	Session two
**Zaidi–Dayal scale**		
Round	1 (6)	0 (0)
Oval	**6 (38)**	3 (19)
Egg-shaped	2 (13)	**7 (44)**
Tetragonal	0 (0)	1 (6)
Pentagonal	0 (0)	0 (0)
Hexagonal	5 (31)	1 (6)
Irregular	2 (13)	4 (25)
**Richards–Jabbour scale**		
Circular	1 (6)	0 (0)
Two semicircles	**6 (38)**	1 (6)
Heart-like	0 (0)	0 (0)
Wide oval	4 (25)	2 (13)
Bi-rounded oval	0 (0)	1 (6)
Ventrally wide oval	1 (6)	2 (13)
Bi-pointed oval	3 (19)	**10 (63)**
Dorsally convergent oval	1 (6)	0 (0)

19

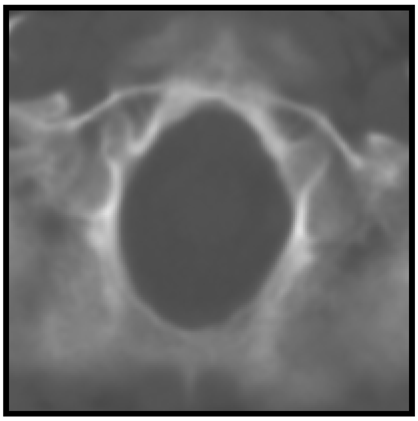

Category	Ratings • *n* (%)	
	Session one	Session two
**Zaidi–Dayal scale**		
Round	0 (0)	0 (0)
Oval	0 (0)	6 (38)
Egg-shaped	**16 (100)**	**8 (50)**
Tetragonal	0 (0)	0 (0)
Pentagonal	0 (0)	0 (0)
Hexagonal	0 (0)	0 (0)
Irregular	0 (0)	2 (13)
**Richards–Jabbour scale**		
Circular	0 (0)	0 (0)
Two semicircles	1 (6)	3 (19)
Heart-like	0 (0)	0 (0)
Wide oval	5 (31)	1 (6)
Bi-rounded oval	3 (19)	4 (25)
Ventrally wide oval	0 (0)	1 (6)
Bi-pointed oval	**7 (44)**	0 (0)
Dorsally convergent oval	0 (0)	**7 (44)**

20

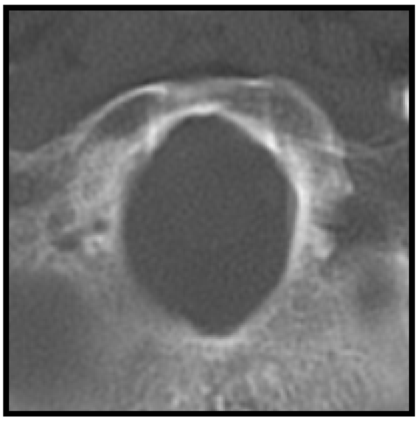

Category	Ratings • *n* (%)	
	Session one	Session two
**Zaidi–Dayal scale**		
Round	0 (0)	0 (0)
Oval	**7 (44)**	**10 (63)**
Egg-shaped	3 (19)	0 (0)
Tetragonal	1 (6)	1 (6)
Pentagonal	0 (0)	0 (0)
Hexagonal	2 (13)	3 (19)
Irregular	3 (19)	2 (13)
**Richards–Jabbour scale**		
Circular	0 (0)	0 (0)
Two semicircles	1 (6)	0 (0)
Heart-like	0 (0)	0 (0)
Wide oval	1 (6)	1 (6)
Bi-rounded oval	2 (13)	1 (6)
Ventrally wide oval	0 (0)	3 (19)
Bi-pointed oval	**9 (56)**	**11 (69)**
Dorsally convergent oval	3 (19)	0 (0)

21

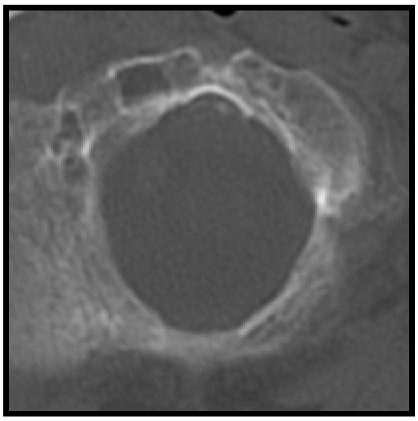

Category	Ratings • *n* (%)	
	**Session one**	**Session two**
**Zaidi–Dayal scale**		
Round	0 (0)	2 (13)
Oval	2 (13)	2 (13)
Egg-shaped	2 (13)	**7 (44)**
Tetragonal	1 (6)	1 (6)
Pentagonal	**8 (50)**	2 (13)
Hexagonal	3 (19)	0 (0)
Irregular	0 (0)	2 (13)
**Richards–Jabbour scale**		
Circular	1 (6)	2 (13)
Two semicircles	**7 (44)**	3 (19)
Heart-like	1 (6)	0 (0)
Wide oval	0 (0)	2 (13)
Bi-rounded oval	2 (13)	0 (0)
Ventrally wide oval	1 (6)	**4 (25)**
Bi-pointed oval	3 (19)	1 (6)
Dorsally convergent oval	1 (6)	**4 (25)**

22

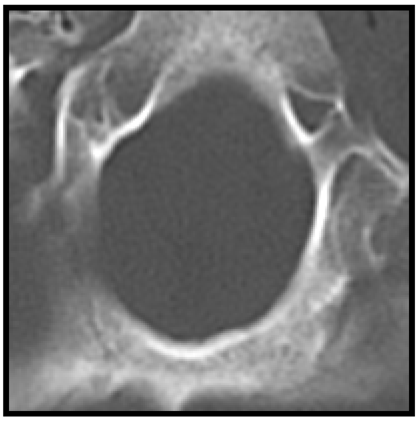

Category	Ratings • *n* (%)	
	Session one	Session two
**Zaidi–Dayal scale**		
Round	0 (0)	0 (0)
Oval	3 (19)	0 (0)
Egg-shaped	4 (25)	5 (31)
Tetragonal	0 (0)	0 (0)
Pentagonal	4 (25)	1 (6)
Hexagonal	**5 (31)**	4 (25)
Irregular	0 (0)	**6 (38)**
**Richards–Jabbour scale**		
Circular	0 (0)	1 (6)
Two semicircles	2 (13)	1 (6)
Heart-like	0 (0)	2 (13)
Wide oval	**7 (44)**	**4 (25)**
Bi-rounded oval	5 (31)	3 (19)
Ventrally wide oval	1 (6)	0 (0)
Bi-pointed oval	1 (6)	3 (19)
Dorsally convergent oval	0 (0)	2 (13)

23

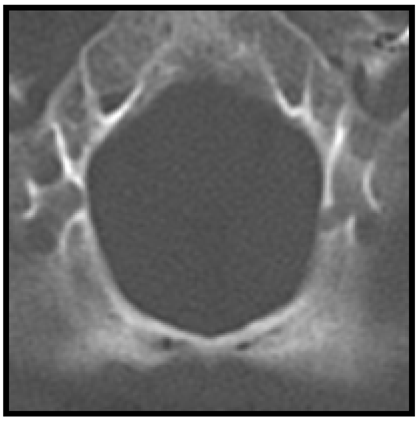

Category	Ratings • *n* (%)	
	Session one	Session two
**Zaidi–Dayal scale**		
Round	1 (6)	2 (13)
Oval	0 (0)	0 (0)
Egg-shaped	0 (0)	0 (0)
Tetragonal	0 (0)	1 (6)
Pentagonal	2 (13)	3 (19)
Hexagonal	**13 (81)**	**10 (63)**
Irregular	0 (0)	0 (0)
**Richards–Jabbour scale**		
Circular	1 (6)	**3 (19)**
Two semicircles	1 (6)	**3 (19)**
Heart-like	1 (6)	1 (6)
Wide oval	0 (0)	1 (6)
Bi-rounded oval	3 (19)	1 (6)
Ventrally wide oval	3 (19)	1 (6)
Bi-pointed oval	2 (13)	**3 (19)**
Dorsally convergent oval	**5 (31)**	**3 (19)**

24

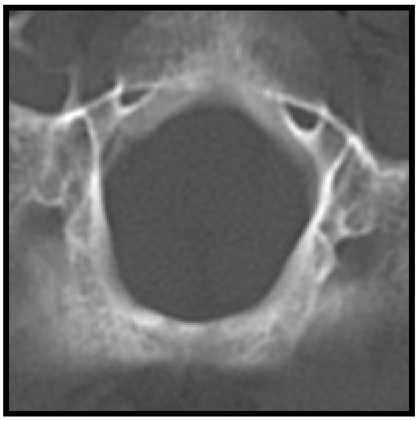

Category	Ratings • *n* (%)	
	Session one	Session two
**Zaidi–Dayal scale**		
Round	1 (6)	0 (0)
Oval	0 (0)	0 (0)
Egg-shaped	3 (19)	2 (13)
Tetragonal	1 (6)	1 (6)
Pentagonal	**11 (69)**	**12 (75)**
Hexagonal	0 (0)	1 (6)
Irregular	0 (0)	0 (0)
**Richards–Jabbour scale**		
Circular	3 (19)	0 (0)
Two semicircles	**4 (25)**	**6 (38)**
Heart-like	1 (6)	1 (6)
Wide oval	2 (13)	3 (19)
Bi-rounded oval	1 (6)	1 (6)
Ventrally wide oval	2 (13)	3 (19)
Bi-pointed oval	1 (6)	0 (0)
Dorsally convergent oval	2 (13)	2 (13)

25

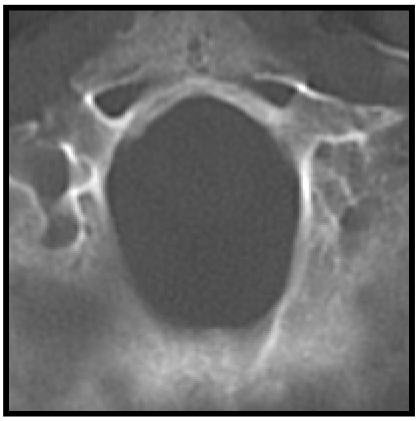

Category	Ratings • *n* (%)	
	Session one	Session two
**Zaidi–Dayal scale**		
Round	0 (0)	0 (0)
Oval	0 (0)	**6 (38)**
Egg-shaped	3 (19)	4 (25)
Tetragonal	0 (0)	0 (0)
Pentagonal	**13 (81)**	**6 (38)**
Hexagonal	0 (0)	0 (0)
Irregular	0 (0)	0 (0)
**Richards–Jabbour scale**		
Circular	0 (0)	0 (0)
Two semicircles	2 (13)	3 (19)
Heart-like	1 (6)	1 (6)
Wide oval	**6 (38)**	**6 (38)**
Bi-rounded oval	1 (6)	2 (13)
Ventrally wide oval	**6 (38)**	3 (19)
Bi-pointed oval	0 (0)	0 (0)
Dorsally convergent oval	0 (0)	1 (6)

26

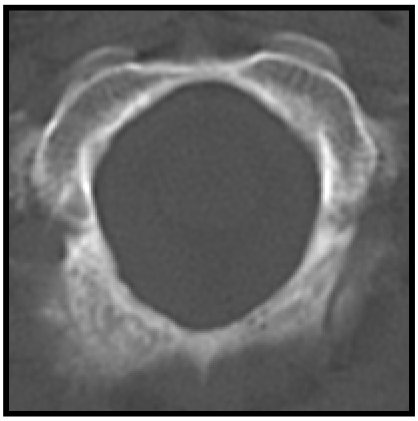

Category	Ratings • *n* (%)	
	Session one	Session two
**Zaidi–Dayal scale**		
Round	2 (13)	2 (13)
Oval	4 (25)	2 (13)
Egg-shaped	2 (13)	**3 (19)**
Tetragonal	0 (0)	**3 (19)**
Pentagonal	0 (0)	1 (6)
Hexagonal	**7 (44)**	**3 (19)**
Irregular	1 (6)	2 (13)
**Richards–Jabbour scale**		
Circular	1 (6)	0 (0)
Two semicircles	3 (19)	**6 (38)**
Heart-like	2 (13)	0 (0)
Wide oval	3 (19)	1 (6)
Bi-rounded oval	1 (6)	3 (19)
Ventrally wide oval	**5 (31)**	1 (6)
Bi-pointed oval	1 (6)	4 (25)
Dorsally convergent oval	0 (0)	1 (6)

27

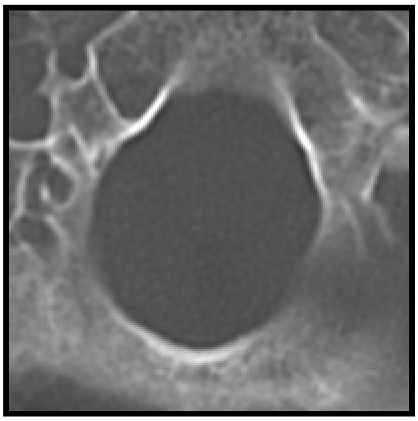

Category	Ratings • *n* (%)	
	Session one	Session two
**Zaidi–Dayal scale**		
Round	3 (19)	4 (25)
Oval	**6 (38)**	1 (6)
Egg-shaped	1 (6)	1 (6)
Tetragonal	0 (0)	1 (6)
Pentagonal	0 (0)	0 (0)
Hexagonal	0 (0)	0 (0)
Irregular	**6 (38)**	**9 (56)**
**Richards–Jabbour scale**		
Circular	2 (13)	4 (25)
Two semicircles	**3 (19)**	1 (6)
Heart-like	1 (6)	0 (0)
Wide oval	**3 (19)**	3 (19)
Bi-rounded oval	**3 (19)**	1 (6)
Ventrally wide oval	**3 (19)**	1 (6)
Bi-pointed oval	0 (0)	0 (0)
Dorsally convergent oval	1 (6)	**6 (38)**

28

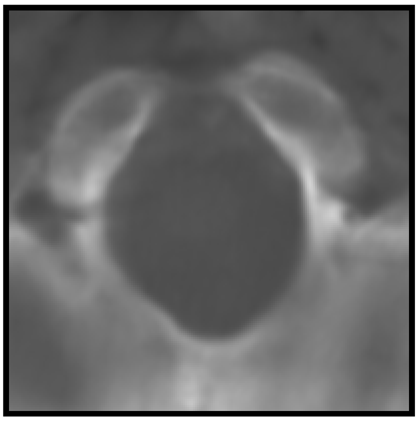

Category	Ratings • *n* (%)	
	Session one	Session two
**Zaidi–Dayal scale**		
Round	0 (0)	0 (0)
Oval	3 (19)	1 (6)
Egg-shaped	4 (25)	3 (19)
Tetragonal	1 (6)	1 (6)
Pentagonal	0 (0)	0 (0)
Hexagonal	**5 (31)**	**7 (44)**
Irregular	3 (19)	4 (25)
**Richards–Jabbour scale**		
Circular	0 (0)	0 (0)
Two semicircles	1 (6)	0 (0)
Heart-like	0 (0)	0 (0)
Wide oval	3 (19)	3 (19)
Bi-rounded oval	3 (19)	3 (19)
Ventrally wide oval	2 (13)	0 (0)
Bi-pointed oval	**6 (38)**	**9 (56)**
Dorsally convergent oval	1 (6)	1 (6)

29

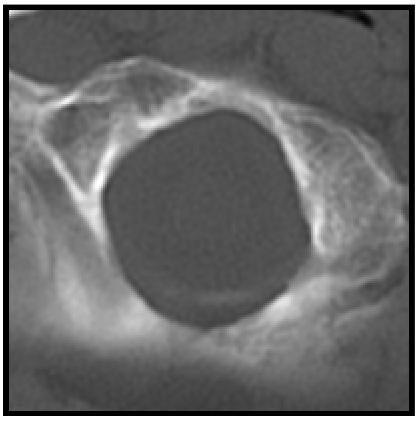

Category	Ratings • *n* (%)	
	Session one	Session two
**Zaidi–Dayal scale**		
Round	3 (19)	4 (25)
Oval	4 (25)	0 (0)
Egg-shaped	**5 (31)**	5 (31)
Tetragonal	1 (6)	0 (0)
Pentagonal	2 (13)	0 (0)
Hexagonal	0 (0)	0 (0)
Irregular	1 (6)	**7 (44)**
**Richards–Jabbour scale**		
Circular	3 (19)	**6 (38)**
Two semicircles	**4 (25)**	4 (25)
Heart-like	0 (0)	0 (0)
Wide oval	**4 (25)**	0 (0)
Bi-rounded oval	1 (6)	3 (19)
Ventrally wide oval	2 (13)	1 (6)
Bi-pointed oval	1 (6)	1 (6)
Dorsally convergent oval	1 (6)	1 (6)

30

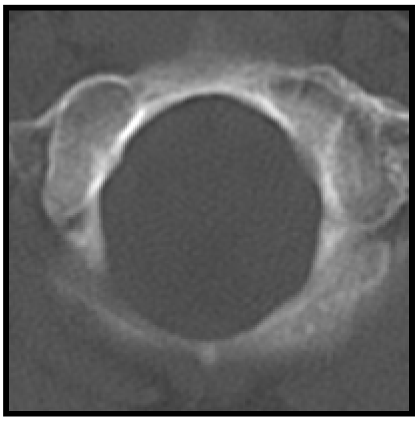

Category	Ratings • *n* (%)	
	Session one	Session two
**Zaidi–Dayal scale**		
Round	**10 (63)**	**9 (56)**
Oval	4 (25)	3 (19)
Egg-shaped	1 (6)	0 (0)
Tetragonal	0 (0)	0 (0)
Pentagonal	0 (0)	2 (13)
Hexagonal	0 (0)	2 (13)
Irregular	1 (6)	0 (0)
**Richards–Jabbour scale**		
Circular	**8 (50)**	**8 (50)**
Two semicircles	7 (44)	7 (44)
Heart-like	0 (0)	0 (0)
Wide oval	1 (6)	0 (0)
Bi-rounded oval	0 (0)	0 (0)
Ventrally wide oval	0 (0)	1 (6)
Bi-pointed oval	0 (0)	0 (0)
Dorsally convergent oval	0 (0)	0 (0)

31

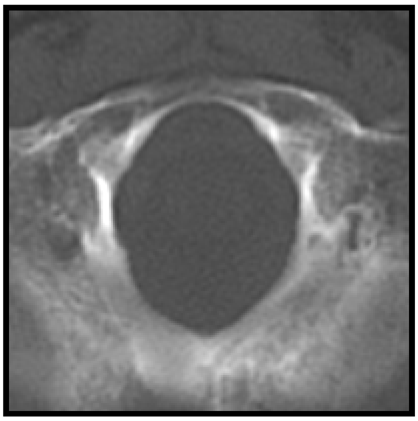

Category	Ratings • *n* (%)	
	**Session one**	**Session two**
**Zaidi–Dayal scale**		
Round	0 (0)	0 (0)
Oval	**12 (75)**	**14 (88)**
Egg-shaped	2 (13)	0 (0)
Tetragonal	1 (6)	0 (0)
Pentagonal	0 (0)	0 (0)
Hexagonal	0 (0)	0 (0)
Irregular	1 (6)	2 (13)
**Richards–Jabbour scale**		
Circular	0 (0)	0 (0)
Two semicircles	3 (19)	1 (6)
Heart-like	1 (6)	0 (0)
Wide oval	3 (19)	**5 (31)**
Bi-rounded oval	1 (6)	**5 (31)**
Ventrally wide oval	0 (0)	1 (6)
Bi-pointed oval	3 (19)	3 (19)
Dorsally convergent oval	**5 (31)**	1 (6)

32

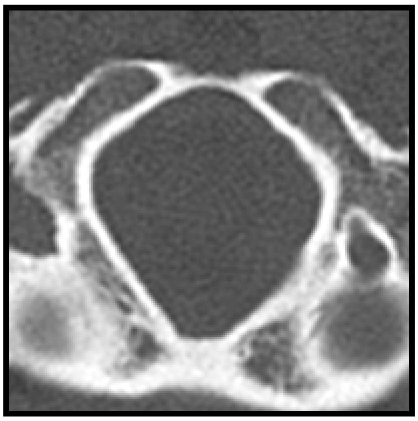

Category	Ratings • *n* (%)	
	Session one	Session two
**Zaidi–Dayal scale**		
Round	0 (0)	0 (0)
Oval	0 (0)	2 (13)
Egg-shaped	1 (6)	0 (0)
Tetragonal	**13 (81)**	**13 (81)**
Pentagonal	2 (13)	0 (0)
Hexagonal	0 (0)	0 (0)
Irregular	0 (0)	0 (0)
**Richards–Jabbour scale**		
Circular	0 (0)	0 (0)
Two semicircles	0 (0)	0 (0)
Heart-like	5 (31)	**6 (38)**
Wide oval	0 (0)	0 (0)
Bi-rounded oval	0 (0)	1 (6)
Ventrally wide oval	5 (31)	1 (6)
Bi-pointed oval	0 (0)	3 (19)
Dorsally convergent oval	**6 (38)**	5 (31)

33

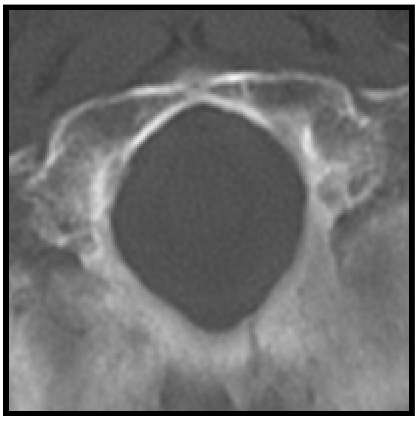

Category	Ratings • *n* (%)	
	Session one	Session two
**Zaidi–Dayal scale**		
Round	2 (13)	1 (6)
Oval	3 (19)	**8 (50)**
Egg-shaped	1 (6)	2 (13)
Tetragonal	**10 (63)**	3 (19)
Pentagonal	0 (0)	0 (0)
Hexagonal	0 (0)	0 (0)
Irregular	0 (0)	2 (13)
**Richards–Jabbour scale**		
Circular	2 (13)	1 (6)
Two semicircles	2 (13)	1 (6)
Heart-like	2 (13)	0 (0)
Wide oval	1 (6)	0 (0)
Bi-rounded oval	1 (6)	3 (19)
Ventrally wide oval	1 (6)	4 (25)
Bi-pointed oval	**4 (25)**	**5 (31)**
Dorsally convergent oval	3 (19)	2 (13)

34

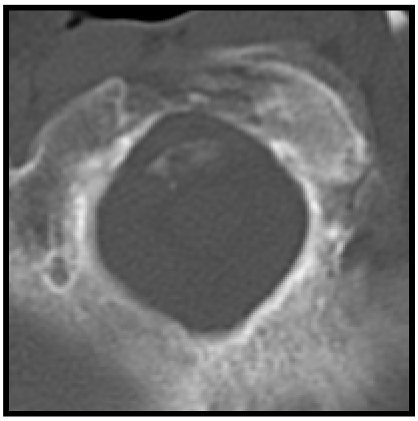

Category	Ratings • *n* (%)	
	Session one	Session two
**Zaidi–Dayal scale**		
Round	0 (0)	0 (0)
Oval	4 (25)	2 (13)
Egg-shaped	0 (0)	2 (13)
Tetragonal	**12 (75)**	**10 (63)**
Pentagonal	0 (0)	2 (13)
Hexagonal	0 (0)	0 (0)
Irregular	0 (0)	0 (0)
**Richards–Jabbour scale**		
Circular	3 (19)	0 (0)
Two semicircles	4 (25)	**7 (44)**
Heart-like	1 (6)	0 (0)
Wide oval	0 (0)	1 (6)
Bi-rounded oval	1 (6)	2 (13)
Ventrally wide oval	0 (0)	0 (0)
Bi-pointed oval	**5 (31)**	6 (38)
Dorsally convergent oval	2 (13)	0 (0)

35

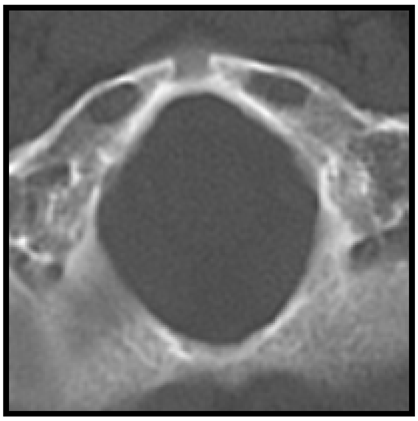

Category	Ratings • *n* (%)	
	Session one	Session two
**Zaidi–Dayal scale**		
Round	0 (0)	0 (0)
Oval	2 (13)	0 (0)
Egg-shaped	**10 (63)**	**10 (63)**
Tetragonal	3 (19)	2 (13)
Pentagonal	0 (0)	3 (19)
Hexagonal	0 (0)	0 (0)
Irregular	1 (6)	1 (6)
**Richards–Jabbour scale**		
Circular	0 (0)	0 (0)
Two semicircles	**4 (25)**	**3 (19)**
Heart-like	1 (6)	2 (13)
Wide oval	**4 (25)**	**3 (19)**
Bi-rounded oval	1 (6)	2 (13)
Ventrally wide oval	1 (6)	2 (13)
Bi-pointed oval	2 (13)	1 (6)
Dorsally convergent oval	3 (19)	**3 (19)**
